# Sleep patterns and psychosocial health of parents of preterm and full-born infants: a prospective, comparative, longitudinal feasibility study

**DOI:** 10.1186/s12884-022-04862-1

**Published:** 2022-07-06

**Authors:** Gunhild Nordbø Marthinsen, Sølvi Helseth, Milada Småstuen, Bjørn Bjorvatn, Signe Marie Bandlien, Liv Fegran

**Affiliations:** 1 Department of Health and Nursing Science, Faculty of Health and Sport Sciences, University of Agder, PO Box 422, 4604 Kristiansand, Norway; 2 Department of Nursing and Health Promotion, Faculty of Health Sciences, Oslo Metropolitan University, Oslo, Norway; 3 Department of Global Public Health and Primary Care, University of Bergen, Bergen, Norway; 4 Section on Neonatology, Department of Pediatrics, Rikshospitalet, Oslo University Hospital, Oslo, Norway

**Keywords:** Feasibility, Longitudinal, Parental sleep, Preterm, Full-born, Postpartum, Health, Nurses, Psychosocial health, Health related quality of life

## Abstract

**Background:**

The early birth and hospitalization of a preterm infant in neonatal intensive care unit can produce several emotional and behavioural responses including sleep problems for parents. Few studies have explored sleep and its associations with health and HRQoL over time in this vulnerable parent population. This purpose of this study was to evaluate the feasibility of a prospective, comparative, longitudinal study of the sleep patterns and psychosocial health of preterm and full-born infants’ parents during the first postpartum year.

**Methods:**

A prospective, comparative, longitudinal feasibility study was conducted. Parents of preterm infants were compared to parents of full-born infants to identify if there were differences in outcomes between the groups. The parents were instructed to wear actigraphs and complete sleep diaries for two consecutive weeks, and responded to a digital questionnaire covering stress, insomnia, fatigue, depression, social support, self-efficacy, and health-related quality of life. Survey data were collected at infant ages of 2, 6, and 12 months, actigraphy and sleep diary data were collected at infant age of 2 months only. Descriptive analysis was used to describe recruitment and attrition rates. Differences between completers and dropouts were analysed with a chi-square test (categorical data) and Mann–Whitney–Wilcoxon test for two independent samples (continuous variables).

**Results:**

Between June 2019 and March 2020, 25 parents of a preterm infant and 78 parents of a full-born infant were recruited from four neonatal intensive care units and two maternity wards, respectively, in four Norwegian hospitals. Feasibility was predefined as recruiting ≥ 75 parents each of preterm and full-born infants. The target for the full-born group was reached. However, the preterm group recruitment was challenging. Actigraphs, sleep diaries, and questionnaires were evaluated as feasible for use in a future study. Attrition rates were high in both groups at 6 and 12 months. No parent-related characteristics were associated with participation at 6 months. At 12 months, dropouts had a statistically significantly lower age in the full-born group (both parents) and higher age and body mass index in the preterm group (fathers).

**Conclusions:**

A longitudinal study is feasible; however, procedural changes, including using active methods and contacting participants, are necessary to increase the recruitment of preterm infants’ parents.

**Supplementary Information:**

The online version contains supplementary material available at 10.1186/s12884-022-04862-1.

## Background

Sleep affects physical and mental health outcomes [1, 2]. Restorative sleep is important for physical, cognitive, and psychological well-being [3]. In the literature, six to eight hours of sleep have been associated with the lowest risk of mortality in healthy adults [3]. The “postpartum period” refers to the period from childbirth to up to six months or until the infant sleeps through the night [2]. During the first three months, parental sleep patterns can be greatly disturbed and lead to daytime sleepiness and fatigue [4]. An unpredictable infant sleep pattern, night-time feedings, and maternal hormonal changes are common causes of sleep disturbances [5]. Despite large variations among infants, most achieve a stabilised sleep pattern at 6 months, and at 12 months, most infants sleep through the night [6].

Preterm births are “births that occur before 37 weeks of gestation” [7]. Fifteen million infants are born prematurely every year worldwide [8]. Despite advances in medical care, preterm births still represent a leading cause of infant mortality and mortality [8]. Parents of preterm infants have described early birth as a traumatic life experience accompanied by long-lasting emotional stress and anxiety [9]. High levels of daily stress can adversely affect parental sleep [10, 11]. Sleep quantity and quality of new mothers of preterm infants have been described as poor in the early postpartum phase, even though they sleep at home and do not participate in the care of the preterm infant [12].

Sleep affects many domains of life [13]. The relationship between sleep and health has been described as bidirectional: poor sleep can increase the severity of health conditions, and the same conditions can adversely affect sleep [14]. “Psychosocial factors” are “psychological sensations or experiences” related to an individual’s physical and social status [15]. Sleep and psychosocial factors are often closely linked [16–18]. Stress is the most consistent factor associated with poor sleep after a preterm birth [11, 19]. Stress adversely affects sleep and has therefore been associated with increased fatigue, anxiety, depression, and reduced HRQoL [10, 12, 20].

Two previous longitudinal cohort studies compared sleep outcomes between mothers of preterm and full-born infants up to four [21] and five months postpartum [22] and reported incongruent results. Gennaro and Fehder [21] did not find any differences in sleep quantity between mothers of preterm and full-born infants, while McMillen et al. [22] reported that mothers of preterm infants had a shorter sleep duration compared to mothers of full-born infants. Neither of these studies included associations between sleep and psychosocial health–related variables or presented data collected over longer periods to assess possible long-term differences between the two groups. To the best of our knowledge, no study has explored sleep and its associations with health and HRQoL over time in this vulnerable parent population [19, 20].

Sleep can be studied either objectively or subjectively using self-reported measures [23]. Actigraphs are small monitors with high reliability to objectively assess circadian rhythm [24]. Sleep diaries are self-reports of sleep and are usually used to measure sleep parameters over days or weeks [25]. Actigraphs and sleep diaries are commonly used in modern sleep research, possessing high accuracy and validity to assess sleep patterns in epidemiological studies [24, 26]. Still, no study has evaluated the feasibility of using such measures in longitudinal studies involving parents of preterm and full-born infants [20].

A feasibility study can provide valuable insights into parts of a future project [27] by answering the following questions: Can it be done? Should we proceed with it? If so, how? [28]. A recent study described the recruitment of preterm infants’ parents as a challenge, as the frequent transportation of infants between hospitals and wards can affect recruitment opportunities [29]. Poor recruitment can threaten internal and external validity in research [30]. An understanding of barriers regarding recruitment and retention in studies can help researchers to develop strategies to overcome these issues [30]. The overall aim of the study was to evaluate the feasibility of a prospective, comparative, longitudinal study of the sleep and psychosocial health of preterm and full-born infants’ parents during the first postpartum year. The primary aim was to assess recruitment and attrition rates. The secondary aims were to 1) describe and compare the characteristics of the participants, 2) evaluate measures and outcomes, and 3) identify possible associations between the selected variables and the attrition rates.

## Methods

### Study design

A feasibility longitudinal study with a case–control design was conducted to meet this study’s aims. Sleep and health-related outcomes from two parent groups (parents of preterm and full-born infants) were evaluated and compared prospectively, with preplanned assessment points at 2, 6, and 12 months after birth.

### Participants

#### Sample size consideration

This feasibility study aimed to recruit ≥ 75 parent couples with a preterm infant and ≥ 75 parent couples with a full-born infant. The sample size was anticipated to be achievable within a limited period – between June and December 2019. The sample size was estimated using data from two previous studies reporting on differences in total sleep time [11, 31]. Mothers of preterm infants slept on average 6.3 (SD 2) hours per night [11], compared to 7.0 (SD 1) hours in a group of mothers of full-born babies [31]. We assumed there was a one-hour difference in the total sleep time between the groups. To account for multiple testing, we estimated that it would be sufficient to include ≥ 75 couples in both groups.

### Recruitment

Between June 2019 and March 2020, postpartum parent couples were recruited from neonatal intensive care units and maternity wards into two groups: Group A and Group B. Parents of preterm infants (born *before* the 37th week of pregnancy) were included in Group A, while parents of full-born infants (born *after* the 37th week of pregnancy) were included in Group B. Recruitment took place two days per week – on Tuesdays and Thursdays.

Parents of preterm infants (Group A) were recruited from four neonatal intensive care units in hospitals in southeastern Norway. Three of the neonatal intensive care units (Hospitals 1, 2, and 3) were at Level 3c, while the last one was at Level 3b (Hospital 4). In Norway, Level 3c units have the highest medical competence to treat extremely preterm infants down to gestational age 23. Level 3b units have the second highest competence and treat preterm infants from gestational age 26 [32].

Parent couples with a full-born infant (Group B) were recruited from two maternity wards (Hospitals 2 and 4) which treat healthy mothers with uncomplicated births. Eligible parents were over 16 years of age, lived together, and had a sufficient command of a Nordic language (written and oral). Both mother-father parents and same-sex parents were included. Parents were recruited within the first five weeks after birth. Parents were excluded if they had a serious drug addiction (recorded in the patient journal, cf. ICD-10 or DSM-IV), the newborn had serious deformities/or a life-threatening condition that could affect survival, the mother had given birth to multiple infants, or the mother had a condition/diagnosis which made participation in the project ethically challenging (serious, life-affecting health issues). Parents with shift work were excluded, as working at night impacts sleep.

Eligible participants were identified by a dedicated nurse in each hospital department. The nurses received informed consent from parents who wanted to participate. Collaborating nurses had been trained to demonstrate how to use an actigraph and a sleep diary.

### Data collection and measurements

All participants wore actigraphs, completed sleep diaries, and filled out questionnaires at baseline (2 months). After the COVID-19 outbreak in March 2020, actigraphs and sleep diaries could not be distributed due to the risk of spreading the virus. At 6 and 12 months, parents responded to questionnaires only. Participating parents received a postal package containing two preprogrammed Actiwatch 2 actigraphs (AW2; Philips Respironics, Mini-Mitter) and preplotted (dates only) sleep diaries at the infant ages of 2 months. Actigraphs and sleep diaries were returned to the first author in a prepaid envelope. In addition, each parent responded to a digital questionnaire composed of questions about insomnia, HRQoL, self-efficacy, depression, social support, fatigue, and stress after two weeks with sleep recordings. Baseline sociodemographic data (age, educational level, income, employment status, ethnicity, weight, height, parity) and infant-related data (gestational age at birth, birthweight, length) were collected at the baseline measurement.

### Sleep assessment

Actigraphy was used to monitor sleep–wake activity. The parents were instructed to wear actigraphs and complete sleep diaries for two consecutive weeks. Actigraphy data were downloaded via a computer and processed with Actiware software (version 6.0.9). Actigraphy has been tested and found reliable compared to polysomnography, especially for total sleep time estimates [33]. Parents were instructed to press an “event button” when they went to bed to sleep for the night and when they got out of bed in the morning. The following measures were retrieved from the actigraphs: sleep-onset latency, time in bed, sleep efficiency, wake after sleep onset, total wake time, and total sleep time.

Sleep diaries were used to detect self-reported sleep data. A sleep diary presented by Morin [34] was used. The following measures were reported from the sleep diary: number of daytime naps, daytime nap duration, daytime function (1, *very good*; 5, *very poor*), sleep-onset latency, wake after sleep onset, number of night-time awakenings, early morning awakening, total wake time, total sleep time, time in bed, sleep efficiency, and sleep quality rating (1, *very restless*; 5, *very poor*).

### Self-report questionnaires

Insomnia was assessed with the Bergen Insomnia Scale, a six-item questionnaire [35]. The questionnaire specifies if participants have experienced insomnia symptoms in the last three months based on the updated DSM-5/International Classification of Sleep Disorders-3 [36]. The Bergen Insomnia Scale has demonstrated good psychometric properties [35].

HRQoL was measured using RAND-36, a 36-item questionnaire that assesses 8 subscales with 35 multi-item scales. Both physical and mental health outcomes are scored using the eight subscales [37]. The Norwegian version of RAND-36 has been reported as a valid and reliable instrument for assessing HRQoL [38].

Self-efficacy was assessed with the Generalized Self-Efficacy Scale, a 10-item scale for the assessment of optimistic self-belief in coping with different life challenges [39]. A revised five-item version of the original scale was used in this study. The short form of the Generalized Self-Efficacy Scale has been found valid and reliable [40].

Postpartum depression was measured using the Edinburgh Postnatal Depression Scale, a 10-item questionnaire measuring depressive symptoms experienced over the past seven days [41]. The Norwegian version of the Edinburgh Postnatal Depression Scale is a valid screening instrument for detecting postpartum depression [42].

Social support was assessed with the Duke-University of North Carolina Functional Social Support Questionnaire [43]. The instrument has been validated as reliable in several samples internationally [43, 44]. No Norwegian version of the Functional Social Support Questionnaire exists; therefore, we translated the English version. The version was not validated.

Fatigue was measured with the Chalder Fatigue Questionnaire (CFQ). The original version was revised and is now more widely used to measure the severity of “tiredness” rather than just chronic fatigue syndrome [45]. The CFQ has demonstrated good clinical validity and internal consistency [46]. A Norwegian validated version of the CFQ was used [47].

Stress was assessed using the Perceived Stress Questionnaire, originally developed to measure stress-related disorders in clinical research [48, 49]. The instrument is considered useful in psychosomatic research on stress [50]. The Perceived Stress Questionnaire has been validated as feasible in the assessment of stress in adolescents but not in adult populations in Norway.

This study included several measures to evaluate sleep and health outcomes in parents. The measures were evaluated according to response rate and the feasibility of collecting data on sleep and selected health-related variables. Based on general acceptance, we considered that a response rate ≥ 70% would be feasible to ensure that the data were sufficiently representative of the sample [51]. Compliant data from the actigraphs were defined as ≥ 1 day with ≥ 24 h of daily wear time on the accelerometer, and compliant data from the sleep diaries were defined as ≥ 1 day with ≥ 24 h of response in the diary. The feasibility of the questionnaire was evaluated regarding receiving a response/no response.

### Statistical analysis

Descriptive analysis was used to describe the study sample and feasibility outcomes (recruitment and attrition rates). Categorical data were presented as frequencies and percentages. Continuous data were described using median, range, and minimum and maximum values.

From the sleep diaries and actigraphs, median values obtained for seven days (five weekdays and two weekend days) were analysed. In the case of missing data, the seven days with the least combined missing data from both measures (actigraphy and sleep diary) were used. Possible differences between completers and dropouts were assessed for available baseline variables. We compared the baseline (2 month) and 6-month data, as well as the baseline (2 month) and 12-month data. Categorical data were analysed with a chi-square test. Pairs of continuous variables were compared using a nonparametric Mann–Whitney–Wilcoxon test for two independent samples. IBM SPSS Statistics for Windows version 26 (IBM Corp., Armonk, NY, USA) was used to conduct statistical analysis.

### Validity and reliability

The questionnaires used to collect data on stress [50], fatigue [47], self-efficacy [40], depression [42], insomnia [35], and HRQoL [38] all represent well-validated, standardised measurements for assessing perceived psychosocial health outcomes. Demographic data were collected by questionnaires from the Norwegian Mother and Child Cohort Study [52]. Previous studies have provided evidence on the use of actigraphy to provide consistent objective data on sleep insomnia and circadian rhythm sleep–wake disorders [53]. Sleep diaries have also been well established to provide data on various sleep parameters (bedtime, SOL, and sleep duration; [54].

### Ethical considerations

This study was designed and implemented according to the Declaration of Helsinki and standard, common clinical research principles [55]. The Norwegian Regional Committee for Medical Research Ethics approved the project (reference no. 2018/1025). Research committees at each hospital gave permission to implement the study in the respective wards. Leaders in each department permitted teaching nursing staff and recruiting parents. Informed consent was obtained from all study participants. Participants were informed that they could withdraw at any time during the study without penalty. Only members of the research team had access to the participant data. In this project, none of the refusing participants were asked the reason for their refusal.

## Results

### Feasibility of recruitment

**Fig. 1:**
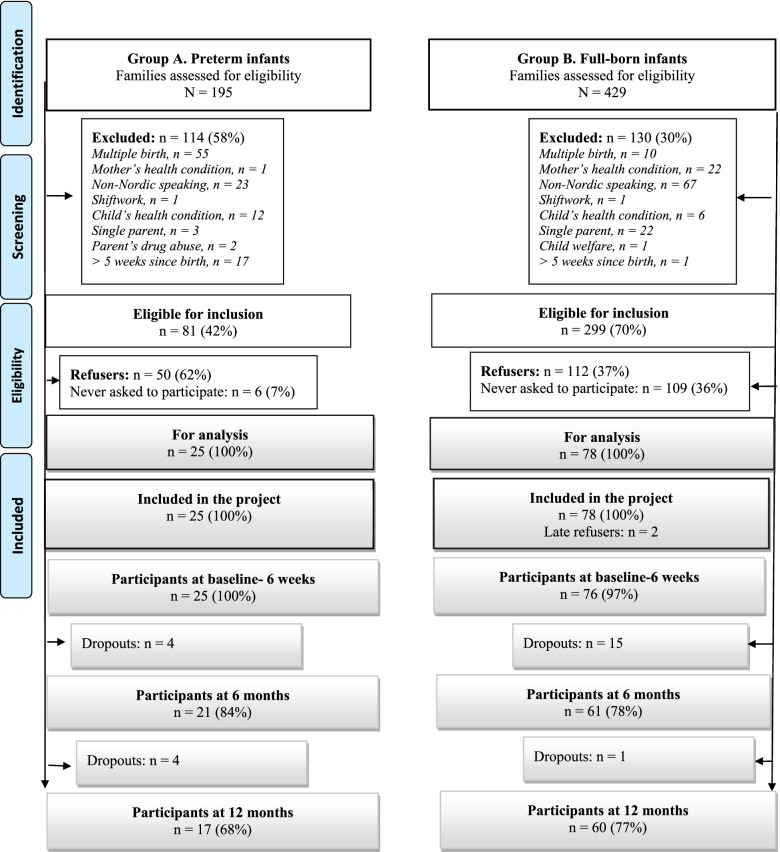
Flow chart of participants

Recruitment was evaluated by calculating a) eligibility rates, the number of parents who met the inclusion criteria was divided by the total number of parents in neonatal intensive care units and maternity wards, and b) consent rates, computed by dividing parents who met the inclusion criteria by the number that consented to participate. Figure 1 illustrates the recruitment process.

### Recruitment of parents of preterm infants (Group A)

In total, 195 parent couples with a preterm infant were screened for participation in the study, of which 114 (58%) were excluded (Fig. 1). The refusal rate was high (62%). In total, 25 couples were finally included in the study, representing a consent rate of 31%. The target set of ≥ 75 couples for this group was never reached. Hospitals permitted different periods to recruit parents. We got permission to recruit for eight weeks each at Hospitals 1 and 2. At Hospital 4, we were permitted to recruit without any time limitation and recruited parents for 22 weeks. The recruitment period was extended past December 2019 due to a low recruitment rate. We also added one more hospital in January 2020 (Hospital 3), where we recruited for three weeks. In March 2020, the Coronavirus disease 2019 (COVID-19) pandemic ended all recruitment efforts.

### Recruitment of parents of full-born infants (Group B)

In total, 429 couples with a full-born infant were screened for participation in the study. Of those, 299 couples were eligible, resulting in an eligibility rate of 70%. A total of 112 couples (37%) refused to participate in the study, resulting in a consent rate of 26%. A total of 109 parent couples were never asked to participate; the high volume of eligible parents overstretched our recruitment capacity. The group consisted of 78 couples. As two couples withdrew their consent before the baseline measurement, 76 couples participated at baseline (Fig. 1). We recruited for 15 (Hospital 1) and 6 (Hospital 2) weeks. The feasibility target was reached by December 2019.

### Attrition rates at 6 and 12 months

Rates of attrition were calculated as participants that completed the questionnaire at 6 and 12 months divided by the number of participants at baseline, 2 months. Table 1 illustrates participants’ completion of measures.

**Table 1:** Completion of measures, 2,6 and 12 months

	**2 months postpartum**		**6 months postpartum**		**12 months postpartum**	
	Mothers	Fathers^c^	Mothers	Fathers^c^	Mothers	Fathers^c^
**Group A Preterm group**						
n (%)	25 (100%)	25 (100%)	21 (100%)	21 (100%)	17 (100%)	17 (100%)
**Questionnaire**						
Responders	16 (64.0)	14 (56.0)	9 (42.9)	6 (28.6)	10 (58.9)	8 (47.0)
Non- responders	9 (36.0)	11 (44.0)	12 (57.1)	15 (71.4)	7 (41.1)	9 (52.9)
**Actigraphy**						
Compliant data^a^	18 (72.0)	18 (72.0)				
Non- compliant data	7 (28.0)	7 (28.0)				
Device error						
**Sleep diary**						
Compliant data^b^	18 (72.0)	18 (72.0)				
Non- compliant data	7 (28.0)	7 (28.0)				
**Group B Full- born group**						
n (%)	76 (100%)	76 (100%)	61 (100%)	61 (100%)	60 (100%)	60 (100%)
**Questionnaire**						
Responders	58 (76.3)	46 (60.5)	39 (63.9)	31 (50.8)	30 (50.0)	31 (51.6)
Non -responders	18 (23.7)	30 (39.4)	22 (36.0)	30 (49.1)	30 (50.0)	29 (48.3)
**Actigraphy**						
Compliant data^a^	53 (69.7)	53 (69.7)				
Non- compliant data	21 (27.6)	23 (30.2)				
Device error	2 (2.6)					
**Sleep diary**						
Compliant data^b^	51 (67.1)	51 (67.1)				
Non- compliant data	25 (32.9)	25 (32.8)				

^a^ Compliant data were defined as ≥ 1 day with ≥ 24 h of daily wear time on accelerometer

^b^ Compliant data were defined as ≥ 1 day with ≥ 24 h of response to sleep diary

^c^ Including Non- birthgiving mothers

### Attrition rates for preterm group (Group A)

A total of nine mothers and six fathers completed the questionnaire at 6 months, producing an attrition rate of 64% for mothers and 76% for fathers. Surprisingly, a few more individuals decided to participate between 6 and 12 months, resulting in attrition rates of 60% (mothers) and 68% (fathers) at 12 months. Two initially nonresponding mothers (baseline) reentered the study at 6 months and one mother reentered at 12 months. Compared to Group B, fathers of preterm infants had the highest attrition rates, with 76% (6 months) and 68% (12 months).

### Attrition rates for full-born group (Group B)

For mothers, the completion rate of the questionnaire was 63.9% at 6 and 50% at 12 months, resulting in attrition rates of 49% (6 months) and 61% (12 months). For fathers, the completion rates were 50.8% (6 months) and 53.4% (12 months), producing attrition rates of 59% at both 6 and 12 months. Similar to preterm group, nonresponders (baseline) reentered the study at 6 and 12 months: one mother and one father reentered at 6, and one father reentered at 12 months.

### Sample evaluation

#### Description and comparison of the participants

Sociodemographic characteristics of the sample (baseline, 2 months) are presented in Tables 2 (parents) and 3 (infants). For parents, there were no large differences between the groups in parental age, body mass index, education, or income level. Most of the parents were primiparas, ethnically Norwegian, and highly educated, and most had an annual income ≥ Norwegian Krone (NOK) 500,000 (≥ €50,000).

**Table 2:** Baseline parent characteristics

	**Group A. Parents of preterm infants**		**Group B. Parents of full-born infants**	
	Mothers, *n* = 25 (100%) (Responders, *n* = 16)	Fathers, *n* = 25 (100%) (Responders, *n* = 14)	Mothers, *n* = 76 (100%) (Responders, *n* = 58)	Fathers^b^, *n* = 76 (100%) (Responders, *n* = 46)
	**Median (min–max)**	**Median (min–max)**	**Median (min–max)**	**Median (min–max)**
**Age**	30.5 (27.0–36.0)	32.0 (27.0–36.0)	31.5 (22.0–42.0)	33.0 (25.0–44.0)
**Body mass index (kg/m**^**2**^**)**	25.1 (19.9–39.8)	25.3.0 (19.6–34.4)	24.4 (19.6–38.6)	26.6 (20.7–35.9)
	**n (%)**	**n (%)**	**n (%)**	**n (%)**
**Ethnicity**				
Norway	15 (93.8)	13 (92.9)	50 (86.2)	42 (91.3)
Nordic Country	1 (6.3)		1 (1.7)	2 (4.4)
Europe			2 (3.4)	1 (2.2)
Asia			2 (3.4)	
South-America		1 (7.1)	2 (3.4)	1 (2.2)
Africa			1 (1.7)	
**Parity**				
0^a^	12 (75.0)	11 (78.6)	38 (65.5)	29 (63.0)
1	4 (25.0)	3 (21.4)	14 (24.1)	10 (21.7)
2			5 (8.6)	6 (13.0)
3			1 (1.7)	1 (2.2)
**Education (level/years)**				
Primary school (up to 10 years)				1 (2.2)
Secondary school (up to 13 years)	4 (25.0)	7 (50.0)	10 (17.0)	9 (20.0)
University/college (up to 4 years)	3 (18.8)	4 (28.6)	22 (37.9)	12 (26.1)
University/college (> 4 years)	8 (50.0)	3 (21.4)	26 (44.8)	22 (47.8)
Other	1 (6.3)			2 (4.3)
**Annual income (NOK)**				
0–399,000	5 (31.3)	3 (21.4)	12 (20.7)	7 (15.6)
400,000–499,000	3 (18.8)	3 (21.4)	18 (31.0)	4 (8.7)
≥ 500,000	8 (50.0)	8 (57.1)	28 (48.3)	35 (76.1)
**Employment status**				
Non-income-generating work situation	1 (6.3)	2 (14.3)	6 (10.3)	3 (6.5)
Income-generating work situation	15 (93.8)	12 (85.7)	52 (89.7)	43 (93.5)
**Missing (nonresponders)**	9 (36.0)	11 (44.0)	18 (23.7)	30 (38.3)

^a^ First-time parents (primiparas)

^b^ Including non-birth-giving mothers

The baseline characteristics of the infants showed that most of the preterm infants were in the least serious preterm category, with most categorised as with a moderate/late gestational level (GA: 32–36) (Table 3). Only one infant was categorised as “extremely preterm (GA < 28),” and only one was “very preterm” (GA: 28–31). According to infant birthweight, seven preterm infants had “normal” birthweight or higher (≥ 2,500- 4,199 g), nine had “low” birthweight (1,500–2,499). Only one had an “extremely” low birthweight (≤ 999 g). For the full-born group, 57 infants had “normal” birthweight (≥ 2,500–4,199 g), and 4 had “high” birthweight (≥ 4,200 g).

**Table 3:** Baseline infant characteristics

	**Group A. Preterm group**, *n* = 25 (100%) (Responders, *n* = 17)	**Group B. Full-born group**, *n* = 76 (100%) (Responders, *n* = 61)
	**n (%)**	**n (%)**
**Infant gestational age (GA)**		
Extremely preterm (GA < 28)	1 (4.0)	–
Very preterm (GA: 28–31)	1 (4.0)	–
Moderate/Late preterm (GA: 32–36)	15 (60.0)	–
Term born (GA ≥ 37)	–	61 (80.0)
Missing	8 (32.0)	15 (19.7)
**Infant birthweight**		
Extremely low (≤ 999 g)	1 (4.0)	–
**Flow chart of participants**		
Very low (1,000–1,499 g)	–	–
Low (1,500–2,499 g)	9 (36.0)	–
Normal weight (≥ 2,500–4,199 g)	7 (28.0)	57 (75.0)
High (≥ 4,200 g)	–	4 (5.2)
Missing	8 (32.0)	15 (19.7)

Note: GA, gestational age

### Representativeness of the sample

#### Parents

The representativeness of the parent sample was evaluated by comparing their sociodemographic characteristics regarding education with those of the general Norwegian population for the same geographic area (Oslo and Sorlandet). Data were obtained from Statistics Norway https://www.ssb.no/en/ , and the 2019 numbers were compared with the distribution of background variables in our sample.

Regarding the level of education, the proportion of individuals in preterm group with less than four years at university (here coded as the highest level of education) was high. Half of the mothers (50%) and one-fifth of the fathers in our sample were highly educated. For the full- born group, 44.8% (mothers) and 48.9% (fathers) had achieved the highest level of education. Fathers in preterm group had a slightly lower proportion (21.4%) of individuals with higher education compared to those in full- born group.

When compared with the general population, for women, 21.6% and 6.9% had the highest level of education for Oslo and Sorlandet, respectively [56]. For men, the figures were 22.3% and 8.2%, respectively [56]. The level of education in our sample for both preterm and full-born group was much higher compared to the general population from the same geographical region.

When we compared the total family income for the Oslo region, parents in our sample earned slightly less than the median annual income of the general population. The parents in preterm group earned slightly below the median total income for the same area (€76,616 vs €95,770), while full-born group parents earned €86,193 versus €95,770. In the Sorlandet area, the median total family income of preterm group and full-born group was about €86,193, which was equal to the median total income for the region [57]. We consider our sample to be representative of the Norwegian population in terms of socioeconomic class, with possibly a slight bias in relation to lower income for parents of preterm infants from the Oslo area.

#### Infants

In 2019, 3,374 infants were born prematurely in Norway [58]. Of those, 754 infants (22%) were born at Hospitals 1 and 2, 171 (5%) at Hospital 4, and 206 (6%) at Hospital 3. The distribution of preterm infants by gestational level and hospital is presented in Additional file 1.

The distribution of the gestational levels of preterm infants was quite similar between the neonatal intensive care units and wards. Despite this, most infants in our sample were in the upper gestational levels, with birthweight in the upper categories. Thus, we consider our sample to be representative of moderate/late preterm (gestational level ≥ 33) infants with birthweight ≥ 1,500 g.

### Evaluation of measures and outcomes

#### Measures

In summary, the amount of compliant data derived from the actigraphs, and sleep diaries was high (≥ 70%) at baseline for parents in preterm group. Table 1 shows the participants’ completion of measures at baseline and throughout the study. For the full-born group, the number was slightly below 70% (Table 1). Two parents requested additional guidance for completing the sleep diary. Two actigraphs had defects; hence, the data were unusable. Three participants reported that they had skin allergies due to their actigraphs; therefore, the wear time was reduced. Otherwise, no adverse events affected the data collection of sleep. For the questionnaire, the response rate was less than 70% in both groups (except for mothers in full-born group at baseline) at all three measure points (Table 1). At 6 months, the response rate in preterm group decreased significantly, despite the fact that the number of participants that remained in the study was relatively high (Fig. 1).

### Outcomes

The descriptive statistics for sleep (actigraphy and sleep diary) at baseline are presented in Table 4. Sleep data were successfully reported for all selected sleep variables, except for daytime naps (actigraph). Many parents forgot to press the event button to register daytime naps and marked night-time sleep only. Descriptive statistics for selected health variables are presented at all three measure points (2, 6, and 12 months) in Table 5.

**Table 4:** Descriptive variables for subjective and objective sleep at baseline (2 months postpartum) for Groups A and B

	**Group A. Preterm group** **Median (min–max)**		**Group B. Full-born group** **Median (min–max)**	
	**Mothers**	**Fathers**^**a**^	**Mothers**	**Fathers**^**a**^
**Sleep diary**	**Responders** ***n* = 18**	**Responders** ***n* = 18**	**Responders** ***n* = 51**	**Responders** ***n* = 51**
Daytime function (1, *very good*; 5, *very poor*)	2.5 (1.6–3.1)	2.4 (1.4–3.1)	2.1 (1.0–3.9)	2.3 (1.0–3.3)
Sleep quality (1, *very restless*; 5, *very sound*)	3.4 (1.6–4.8)	3.4 (2.0–4.7)	3.0 (1.5–4.6)	3.4 (1.6–4.3)
Number of daytime naps	0.4 (0–0.9)	0.2 (0–0.9)	0.1 (0–1.1)	0.0 (0–0.7)
Daytime nap duration (min)	24.0 (0–62.0)	7.9 (0–70.0)	4.3 (0–60)	0.0 (0–49.0)
Sleep onset latency (min)	10.8 (1–82.0)	12.3 (0–70.0)	31.4 (1–89.0)	13.5 (0–98.0)
Number of nighttime awakenings	2.4 (1.3–4.9)	1.8 (0.6–3.7)	2.7 (0.6–5.0)	1.4 (0–4.7)
Wake after sleep onset (min)	114.3 (12.4–253.8)	29.9 (3–96.0)	71.4 (8.6–155.0)	13.6 (0–74.3)
Early morning awakening (min)	13.1 (5.7–43.3)	9.6 (0–85.0)	15.7 (0.7–72.9)	13.3 (0–51.9)
Total wake time (min)	147.4 (40.0–247.5)	68.6 (30.0–132.7)	125.0 (29.7–237.1)	45.0 (1.6–131.0)
Time in bed (min)	538.2 (421.4–707.9)	475.0 (373.0–621.4)	569.3 (415.4–743.6)	475.7 (346.3–647.1)
Total sleep time (min)	415.1 (301.3–516.4)	411.9 (309.3–507.1)	427.0 (302.9–582.9)	425.7 (274.4–567.0)
Sleep efficiency (%)	72.6 (52.3–91.2)	85.2 (73.9–93.8)	78.1(59.6–93.4)	90.6 (71.8–99.6)
**Actigraph**	**Responders** ***n* = 18**	**Responders** ***n* = 18**	**Responders** ***n* = 53**	**Responders** ***n* = 53**
Time in bed (min)	528.8 (431.2–695.4)	466.0 (336.6–625.6)	538.0 (395.0–702.6)	462.0 (356.6–560.0)
Total sleep time (min)	421.0 (343.4–500.8)	387.4 (292.1–500.9)	433.0 (328.0–561.8)	397.7 (315.1–468.9)
Sleep onset latency (min)	13.1 (2.4–30.6)	15.3 (1.6–59.1)	13.0 (1.0–65.4)	13.0 (2.9–48.2)
Sleep efficiency (%)	78.3 (65.8–87.9)	85.8 (64.7–91.0)	81.5 (66.7–90.1)	85.3 (77.1–91.2)
Wake after sleep onset (min)	100.0 (31–192.2)	37.6 (26.1–88.4)	61.7 (31.5–143.0)	34.0 (14.4–73.0)

^a^ including non-birth-giving mothers

**Table 5:** Descriptive variables for insomnia, depression, fatigue, depression, social support, self-efficacy, stress and HRQoL at 2, 6 and 12 months postpartum for preterm and full- born infants parents

	**2 months postpartum**		**6 months postpartum**		**12 months postpartum**	
	Mothers	Fathers^a^	Mothers	Fathers^a^	Mothers	Fathersa
**Group A. Preterm group**	Responders, *n* = 16	Responders, *n* = 14	Responders, *n* = 9	Responders, *n* = 6	Responders, *n* = 10	Responders, *n* = 8
Insomnia, n (%)	10 (62.5)	10 (71.4)	5 (55.6)	2 (33.3)	5 (50.0)	1 (12.5)
Fatigue, n (%)	9 (56.3)	6 (42.9)	5 (55.6)	1 (16.7)	4 (40.0)	3 (37.5)
Depression, n (%)	2 (12.5)	2 (14.3)	3 (33.3)	–	3 (30.0)	1 (12.5)
HRQoL (median, min/max)						
Physical well-being	49.6 (20.8–64.5)	49.7 (42.8–58.7)	55.1 (40.6–61.5)	52.6 (50.6–55.8)	51.7 (38.4–58.0)	50.0 (41.8–56.4)
Mental well-being	48.7 (32.0–59.2)	48.7 (28.1–58.3)	47.5 (32.2–57.2)	50.7 (43.5–58.0)	50.8 (26.3–66.6)	50.5 (31.2–58.0)
Self-efficacy (median, min/max)	14.5 (8.0–19.0)	16.5 (11.0–20.0)	15.0 (10.0–18.0)	17.0 (13.0–20.0)	14.5 (12.0–20.0)	15.5 (10.0–20.0)
Social support (median, min/max)	1.1 (1.0–2.5)	1.3 (1.0–1.8)	1.3 (1.0–2.4)	1.3 (1.0–2.3)	1.5 (1.0–2.38)	1.6 (1.0–2.6)
Stress (median, min/max)	0.3 (0.1–0.6)	0.3 (0.1–0.6)	0.3 (0.2–0.5)	0.2 (0.1–0.3)	0.3 (0.2–0.6)	0.3 (0.1–0.5)
**Group B. Full-born group**	Responders, *n* = 58	Responders, *n* = 46	Responders, *n* = 39	Responders, *n* = 31	Responders, *n* = 30	Responders, *n* = 31
Insomnia, n (%)	31 (53.4)	20 (4.5)	27 (69.2)	13 (41.9)	20 (66.7)	13 (41.9)
Fatigue, n (%)	37 (63.8)	15 (33.3)	21 (53.8)	13 (41.9)	16 (53.3)	12 (38.7)
Depression, n (%)	11 (19.0)	7 (15.2)	11 (28.2)	4 (12.9)	12 (40.0)	9 (29.0)
HRQoL (median, min/max)						
Physical well-being	50.0 (25.0–65.5)	53.7 (37.0–61.3)	52.6 (50.6–55.8)	54.0 (30.7–65.2)	51.9 (24.1–62.7)	52.7 (40.1–65.5)
Mental well being	50.0 (32.0–59.2)	51.5 (23.8–59.5)	52.3 (21.9–60.0)	52.4 (24.2–59.6)	50.2 (18.8–61.5)	50.5 (18.7–60.6)
Self-efficacy (median, min/max)	16.5 (7.0–20.0)	16.5 (11.0–20.0)	17.0 (9.0–20.0)	16.0 (9.0–20.0)	16.0 (8.0–20.0)	15.0 (9.0–20.0)
Social support (median, min/max)	1.6 (1.0–4.5)	1.6 (1.0–4.6)	1.6 (1.0–4.0)	1.8 (1.0–3.8)	1.8 (1.0–4.0)	1.9 (1.0–4.6)
Stress (median, min/max)	0.4 (0–0.9)	0.3 (0.2–0.9)	0.3 (0.9–0.71)	0.3 (0.4–0.8)	0.4 (0–0.8)	0.3 (0–0.9)

^a^ Including non-birth-giving mothers

Parents in preterm group had lower median total sleep time compared to parents in full-born group, as reported in the sleep diaries. According to the sleep diaries, mothers in preterm group also had the lowest median sleep efficiency (72.6%), followed by mothers in full-born group (78.1%). Fathers in both groups reported sleep efficiency ≥ 85% in the sleep diaries; normal sleep efficiency is considered to be 85% or higher [59]. Similarly, for actigraphy, mothers in preterm group had the lowest median value for sleep efficiency (78.3%), followed by mothers in full-born group (81.5%). Fathers were above the normal sleep efficiency value cut-off (Table 4). When compared, mothers in preterm group showed a tendency to sleep more during the daytime and reported a median daytime nap duration of 24 min (sleep diary) compared to mothers in full-born group. The former group also tended to report the highest wake after sleep onset (median = 114.3 min, sleep diary; 100 min, actigraph) per week. Overall, participants in both groups rated their sleep quality as medium; daytime function was good (Table 4).

For the selected health variables, there was a tendency towards high frequencies of insomnia at baseline. The prevalence remained high, particularly for mothers (Table 5) at follow-ups. Fathers in preterm group reported the highest baseline proportions, with 78.6% at baseline. At 6 and 12 months, mothers showed higher proportions of insomnia compared to fathers. Fatigue level was also high, with mothers reporting higher median values at all three measure points compared to fathers, and the difference remained stable at 6 and 12 months. For HRQoL, there were no large differences between both genders and both groups throughout the study.

### Identification of selected variables associated with attrition rates at 6 and 12 months

#### Participation at 6 months

At 6 months, we did not find any baseline characteristics associated with parents’ participation in preterm or full-born group. Additional files 2 and 3 illustrate participant characteristics associated with completion/dropout at 6 months (see Additional files 2 and 3).

#### Participation at 12 months

At 12 months, maternal dropouts in full-born group were of a significantly lower age (*p* = 0.03) compared to completers. No other characteristics were found to be statistically associated with mothers’ participation. For fathers, we found that higher age (*p* = 0.05) and body mass index (*p* = 0.03) were statistically significantly associated with dropout in preterm group. Conversely, a lower age was statistically associated with dropout in full-born group (*p* = 0.03) at T2. No other characteristics at baseline were associated with participation at 12 months. Additional file 4 and 5 illustrate participant characteristics associated with completion/dropout at 12 months [see Additional file 4 and 5].

## Discussion

This is, to our knowledge, the first study to evaluate the feasibility of a prospective, comparative, longitudinal study of the sleep and psychosocial health of parents of preterm and full-born infants during the first postpartum year. The primary aim was to assess recruitment and attrition rates. The secondary aims were to 1) describe and compare the characteristics of the participants, 2) evaluate measures and outcomes, and 3) identify possible associations between the selected variables and attrition rates.

Based on our findings, a future longitudinal study may be feasible; however, changes are recommended, particularly to optimise recruitment to the preterm group. Efforts to minimise attrition rates at 6 and 12 months are needed. The lessons learned from this feasibility study may be helpful to other researchers planning similar studies.

The recruitment of parents of preterm infants represented a major barrier in this study; the predefined feasibility target of 75 couples was never reached. A low eligibility rate (42%) was a large barrier to the inclusion of parents. The volume of twins/multiple infants was surprisingly high (48.2%) and contributed to many exclusions. Parents of twins and multiple newborns have more severe sleep problems than parents of singletons [60, 61]. For this reason, they were excluded from the study.

Additionally, a high volume of non-Nordic speaking individuals (20%) contributed to a high exclusion rate. Parents without a sufficient command of a Nordic language were not included because there were only Norwegian versions of the information sheets and questionnaires. The child’s health condition was also often poorer for parents of preterm infants, leading to 10.5% of exclusions. Preterm infants are often hospitalised for weeks/months and frequently transported between hospitals for medical care [29]. For such reasons, 14.9% of the preterm infants were excluded because they were too old (> 5 weeks) when we tried to recruit them for the project.

The refusal rate was high (62%) for parents in preterm group, compared to full-born group (37%). Since refusers did not have to provide any reason for their denial, it is difficult to ascertain possible explanations. Infants with low gestational levels have a higher risk of morbidity and mortality [62]. Parents have described mental unpreparedness, stress, and concern for their infant’s health condition [63]. This can affect parents’ willingness to participate in research [64]. To compensate for the low recruitment rate, we prolonged the recruitment period for the preterm group by several months after December 2019. Moreover, an additional neonatal intensive care unit (Hospital 3) was included in January 2020. In March 2020, the COVID-19 pandemic ended all recruitment efforts. By then, only 25 couples with preterm infants had been recruited to preterm group.

In total, 78 parent couples with full-born infants were recruited to full-born group, thus confirming that the feasibility target of 75 couples was attainable. The eligibility rate for this group was high (70%). The most common reason for ineligibility was having insufficient command of a Nordic language and being a single parent (Fig. 1). However, the consent rate was low (26%), indicating a need for support. Childbirth and transition to parenthood is generally considered a stressful period for parents [65]. The low consent rate might have been impacted by such factors. Parents were recruited early after childbirth when the situation was still new and demanding. Postpartum parents are difficult to recruit for research [66–68], and our findings support this.

Failure to recruit study participants is a common problem in research; hence, it is important to identify barriers and overcome them [69]. Our findings reveal a need for changes in the recruitment procedure. For preterm group, a broadening of the inclusion criteria is necessary to include a larger group of parents, particularly considering that many parents with minority backgrounds were excluded. A study from Sweden recently confirmed our results; minority parents and twins were common reasons for the exclusion of parents in a similar project [29]. English and translated versions of questionnaires could be designed to include parents with multiple ethnicities. Understanding how ethnic factors are related to sleep and health is essential to meet the care needs of the parent population [70].

On forehand, we anticipated that parents of preterm infants would be more difficult to recruit since they have a higher burden compared to parents of healthy full-born infants [9]. Parents of extremely preterm infants with low birthweight have described the situation as psychologically demanding due to a higher risk of infant mortality and morbidity [71]. Such circumstances can explain why our sample primarily consisted of mainly moderate/late preterm infants (Table 3). The most vulnerable parents (those of infants with the lowest gestational level) were never recruited for the project, even though we collaborated with large Level 3c units with a high admission of extremely/very preterm infants per year (see Additional file 1). New research participants might be difficult to reach for various reasons [72]. “Hidden” groups can be underrepresented in research with large samples, and the research does not entirely reflect their status [73]. Previous studies have shown that different factors can impact parents participation in research negatively; having infant with lower gestational age, infant illness, lack of socioeconomic support, lower parental education level, race, or lack of intact family [74–78]. Despite that previous research successfully has included parents of preterm infants in several studies; we have identified significant methodological challenges if these parents shall be successfully recruited in future cohort studies. Several adjustments are necessary to increase success of these parents. Standard recruitment strategies may not be appropriate to recruit “hidden subgroups” like e.g., parents with extremely low gestational level. More in-depth knowledge regarding their special situation, with particular focus on mental and physical demands might be required to successfully recruit them to research. Strategies to do research participation less burdensome, and support of families with particular high risk of recruitment and retainment issues may facilitate their participation [79]. Reduction of risks, support of resources, building of trust with participants, and use of flexible and creative recruitment strategies might be examples of ways to support recruitment of vulnerable and “hidden” research participants [80]. Fathers of preterm infants with very low birthweight have reported that a lack of emotional support negatively affects their willingness to be recruited for research [64]. Parents need emotional support from health personnel and require help to cope with the unexpected and demanding situation [81]. More focus on face-to-face consultations, family-centred care, and emotional and practical support can increase recruitment success [64]. Improving health personnel’s competence in addressing parents’ psychosocial needs and strengthening communication skills can also heighten recruitment potential [81].

The recruitment of parents of preterm infants raised many ethical questions for our recruiters (nurses). Parents sometimes experience stress levels that meet the diagnostic criteria for acute stress disorder or posttraumatic stress disorder [82, 83]. If parents had a temporary difficult situation (e.g., related to an uncertain infant health situation), our recruiters postponed the formal request of participation until the next recruitment day. Individuals’ capacity to give valid consent can be affected by their emotional state, degree of understanding, and available time to decide [84]. For the recruitment of parents to be successful in future studies, a close collaboration between researchers and clinical staff is essential [51]. Our collaboration with NICU nurses was important since they had a unique position to observe and follow up postpartum parents’ mental health situation during the hospitalization period. Our recruiting nurses had the ability to contact inside- hospital support like psychologist or mental health services if parents needed such support. Parents were also informed to contact their own doctor or health centre if experiencing mental health challenges after discharge from hospital.

A long recruitment period and collaboration with several neonatal intensive care units in Nordic countries may be necessary to recruit parents of preterm infants in forthcoming projects.

Recruitment to the full-born group was faster, considering the volume of parents willing to participate was higher than expected. The nurses’ capacity was often overstretched, and 109 parent couples were never asked to participate. In future projects, a sufficient number of collaborating nurses is needed to assess the high volume of parents of full-born infants. The high volume of parents of full-born infants also challenged our actigraphy resources. Although actigraphs offer many advantages for sleep research [85], they represented a high cost in this study and later limited our recruitment opportunities. We were also dependent on parents returning the actigraphs after use to have them available for new participants.

The identification of potential barriers is important to support recruitment in the future [30]. Seeking the involvement of nurses in recruitment was a challenge since it added to their daily tasks in the wards. The use of health care personnel as recruiters can be challenging because they often have limited time and a high workload [86, 87]. Similar barriers have been reported in other studies [29]. Future studies should ensure that sufficient resources are available for the recruitment of participants, particularly parents of full-born infants.

Recruiting parents as couples was another barrier. Inclusion demanded consent from both parents, representing a problem if only one parent was present in the ward. Similar barriers have been reported elsewhere [29, 64]. Our recruiters attempted to overcome this by recruiting parents at different times of the day. Information sheets were left for absent parents, and postboxes were hung in the wards to collect consent forms from absent parents.

Failure to recruit a representative sample can result in poor generalisability of results [51]. Our sample of parents was representative of the Norwegian population in terms of socioeconomic class, with a slight bias in relation to lower income for preterm infants from the Oslo area. Similar to other projects, individuals with a lower socioeconomic status, low income, or poor education were underrepresented [30, 51, 88, 89].

This study included various research instruments to assess sleep and health outcomes. Based on our evaluations, all measures were feasible for use in a forthcoming project. There were some minor issues regarding some of the measures used in the study. First, it was noted that some participants, for unknown reasons, had some off-time events during the day. Some actigraphy recordings, therefore, had missing intervals. Previous studies have reported that low wear time is a problem [90]. Our overall evaluation demonstrated that actigraphy, sleep diaries, and questionnaires are feasible for use in a forthcoming study; thus, support of the response rates (sleep diary, baseline for full- born group, and questionnaire) is necessary in the future.

A longitudinal study requires a large number of motivated participants who can commit themselves to the study and long-term follow-up [51]. A long study duration and the use of repeated measures can be experienced as burdensome by participants and increase attrition [89]. “Attrition” refers to the failure of participants to complete their participation after being enrolled in a study [79]. Attrition is associated with a loss of statistical power and the risk of selective attrition bias [30]. Bias is expected in study results if attrition exceeds 20% [91, 92]. In the present study, we did not identify any similar determinants associated with attrition at 6 months. At 12 months, dropouts had a lower age in full- born group (both parents) and a higher age and body mass index in preterm group (fathers). Future projects might benefit from testing methods to minimise attrition of these respective participants. In sum, attrition at 6 and 12 months was high for both groups.

Attrition could sometimes be temporary; individuals reentered the study at 6 and 12 months. Other cohorts have reported similar tendencies [92]. To minimise attrition in general, active methods such as reminder letters and phone calls can be helpful [30]. “Barrier-reducing strategies” are particularly important within longitudinal studies [93, 94]. Difficulties, discomfort, or high demands from research design negatively affect participation [30]. Reduction in participant burden (e.g. from data collection) has especially been highlighted to minimize attrition [93]. Collection of sleep and health data using survey alone could be considered [95]. A long study duration, along with a burdensome data collection procedure, can represent a large barrier for the recruitment and retainment of parents [62, 64]. A study design with less extensive data collection could also be appropriate for some parent subgroups. Qualitative study designs could provide a deeper understanding of sleep and health matters [96] and be a less burdensome approach to studying sleep and health in parents with extremely preterm infants.

### Limitations

This study had strengths and limitations. A lack of demographic data on refusers limited our possibility to observe if certain groups were overrepresented in those who declined to participate. Data on individuals who declined participation can provide valuable information to develop more successful recruitment strategies [97, 98]. Some parents did not respond to the baseline questionnaire, and we did not have sociodemographic and infant data on the entire sample. To increase the amount of data for statistical analyses, compliant data from the actigraphs and sleep diaries were defined as ≥ 1 day with ≥ 24 h of daily wear time. Seeking involvement from hospitals was a time-demanding challenge; approvals from ethical committees and hospital leaders took longer than we anticipated. It was also difficult to find collaborating nurses who were willing to spend time recruiting for the project. The COVID-19 pandemic ended our recruitment efforts of parents of preterm infants; otherwise, recruitment would have continued until the targeted number of parents was reached. The sample size was limited and for categorical variables, some of the categories included too few individuals to make any statistical comparisons possible. The pandemic also prevented adequate assessment of the feasibility of using actigraphs and sleep diaries in the long term. Parents of preterm infants were also more vulnerable than expected; this adversely affected recruitment. Lastly, more resources should have been prioritised for the recruitment of parents of healthy full-born infants to meet the high volume of couples.

The strength of this study was sleep and health assessment over time with extensive data collection, as well as the inclusion of fathers. To our knowledge, no similar study has studied sleep and selected health variables over time using a similar study design [19, 20].

## Conclusions

A feasibility study can provide valuable insight into parts of a future project by asking “whether something can be done, should we proceed with it, and if so, how” [28]. Based on our findings, a future study can be done, but several changes are recommended. Particularly, recruitment and attrition need support. Refinement of the inclusion criteria is important to increase the recruitment of preterm infants’ parents. We suggest including English-speaking parents and minority groups, as well as teaching clinical personnel about recruitment. We also recommend a long recruitment period and cooperation with several neonatal intensive care units in Nordic countries to reach a higher volume of parents. Parents of infants with severe and extremely low gestational levels are vulnerable; hence, we suggest simplifying the data collection procedure and investigating sleep and health outcomes with qualitative study designs. For parents of full-born infants, a focus on sufficient recruitment resources is important. Attrition can be minimized with barrier-reducing efforts. We recommend using active methods such as contacting participants. The findings of this study are important for other researchers planning similar studies with the same parent populations.

## Supplementary Information

**Additional file 1.**

**Additional file 2.**

**Additional file 3.**

**Additional file 4.**

**Additional file 5.**

## Data Availability

The datasets used in this current study is available from the corresponding author on reasonable request.

## Abbreviations

COVID-19: Corona Virus Disease-2019
RAND-36: Rand Medical Outcome Study
BIS: Bergen insomnia scale
GA: Gestational level
SOL: Sleep onset wake after sleep onset
WASO: Wake after sleep onset
TWT: Total wake time
TST: Total sleep time
TIB: Time in bed
SE: Sleep efficiency
