# Supplementary material for: Sleep patterns and psychosocial health of parents of preterm and full-born infants: a prospective, comparative, longitudinal feasibility study
Source: BMC Pregnancy Childbirth. 2022 Jul 6;22:546. doi: 10.1186/s12884-022-04862-1 (PMC9258469; doi:10.1186/s12884-022-04862-1)
Supplement: Supplementary file 1 — Additional file 1. [file 12884_2022_4862_MOESM1_ESM.docx]

**Table S1. All infants born in 2019 by gestational age and hospital**

|  | All infants born in Norway in 2019  n = 55,051 (100%) | All infants born at hospitals 1 and 2  n = 9,631 | Recruited from hospitals 1 and 2  n (A) = 9  n (B) = 38 | All infants born at hospital 4  n = 3,094 | Recruited from  hospital 4  n (A) = 16  n (B) = 40 | All infants born at hospital 3  n = 4,263 | Recruited from hospital 3  n = 0 |
| --- | --- | --- | --- | --- | --- | --- | --- |
|  | **n (%)** | **n (%)** | **n (%)** | **n (%)** | **n (%)** | **n (%)** | **n (%)** |
| Extremely preterm  (GA < 28) | 164 (0.3) | 80 (0.83) |  | 2 (0.1) | 1 (1.7) | 14 (0.3) | – |
| Very preterm  (GA 28–32) | 525 (1.0) | 130 (1.3) | 1 (2.1) | 30 (1.0) |  | 31 (0.7) | – |
| Moderate/late preterm (GA 33–36) | 2,685 (4.9) | 544 (5.6) | 4 (8.5) | 139 (4.5) | 11 (19.6) | 161 (3.8) | – |
| Full-born infants  (GA > 37) | 51,677 (93.9) | 8,877 (92.2) | 29 (61.7) | 2,923 (94.5) | 32 (57.1) | 4,057 (95.2) | – |
| Missing |  |  | n (A) = 4  n (B) = 9 |  | n (A) = 4  n (B) = 8 |  |  |

Note. GA, gestational age; A, the preterm group; B, the full-born group.
