# Supplementary material for: Sleep patterns and psychosocial health of parents of preterm and full-born infants: a prospective, comparative, longitudinal feasibility study
Source: BMC Pregnancy Childbirth. 2022 Jul 6;22:546. doi: 10.1186/s12884-022-04862-1 (PMC9258469; doi:10.1186/s12884-022-04862-1)
Supplement: Supplementary file 2 — Additional file 2. [file 12884_2022_4862_MOESM2_ESM.docx]

**Table S2. Selected variables associated with response/nonresponse at six months postpartum (mothers)**

|  | **Group A. Preterm group**  N = 21 | | | **Group B. Full-born group**  N = 61** | | |
| --- | --- | --- | --- | --- | --- | --- |
|  | Completers  n = 9 (42.9%) | Dropouts  n = 12 (57.1%) | p-value | Completers  n = 39 (63.0%) | Dropouts  n = 22 (36.0%) | p-value |
|  | **n (%)** | **n (%)** |  | **n (%)** | **n (%)** |  |
| Infant’s birthweight | 9 (42.9) | 12 (57.1) | N/A* | 39 (63.0) | 22 (36.0) | 1.0 |
| Infant’s gestational age level | 9 (42.9) | 12 (57.1) | N/A* | 39 (63.0) | 22 (36.0) | N/A* |
| Parity | 9 (42.9) | 12 (57.1) | 0.59 | 39 (63.0) | 22 (36.0) | N/A* |
| Fatigue | 9 (42.9) | 12 (57.1) | 0.36 | 39 (63.0) | 22 (36.0) | 0.1 |
| Depression | 9 (42.9) | 12 (57.1) | 0.48 | 39 (63.0) | 22 (36.0) | 0.7 |
| Insomnia | 9 (42.9) | 12 (57.1) | 0.30 | 39 (63.0) | 22 (36.0) | 0.8 |
| Education | 9 (42.9) | 12 (57.1) | N/A* | 39 (63.0) | 22 (36.0) | N/A* |
| Income | 9 (42.9) | 12 (57.1) | N/A* | 39 (63.0) | 22 (36.0) | N/A* |
| Employment status | 9 (42.9) | 12 (57.1) | 1.0 | 39 (63.0) | 22 (36.0) | 0.4 |
| Ethnicity | 9 (42.9) | 12 (57.1) | 1.0 | 39 (63.0) | 22 (36.0) | N/A* |
|  | **Median (range)** | **Median (range)** |  | **Median (range)** | **Median (range)** |  |
| Body mass index | 26.0 (19.8) | 24.0 (13.9) | 0.6 | 25.0 (19.0) | 23.8 (12.0) | 0.3 |
| Age | 28.0 (6.0) | 31.0 (9.0) | 0.2 | 32.0 (16.0) | 30.5 (18.0) | 0.1 |
| HRQoL (physical) | 50.0 (14.7) | 49.1 (43.7) | 1.0 | 50.1 (40.4) | 49.9 (25.1) | 0.9 |
| HRQoL (mental) | 51.2 (14.8) | 38.4 (27.2) | 0.1 | 49.2 (44.0) | 51.5 (32.2) | 0.9 |
| Stress | 0.3 (0.2) | 0.3 (0.5) | 0.5 | 0.4 (0.6) | 0.4 (0.7) | 0.4 |
| Social support | 1.3 (0.9) | 1.0 (1.5) | 0.6 | 1.6 (2.5) | 1.5 (3.5) | 0.5 |
| Self-efficacy | 14.0 (4.0) | 15.0 (11.0) | 1.0 | 16.0 (13.0) | 17.0 (13.0) | 0.8 |

* N/A = N/A not analysed due to too small sample size/too limited statistical power.

** Including non-birth-giving mothers. Range = max-min value
