# Supplementary material for: Sleep patterns and psychosocial health of parents of preterm and full-born infants: a prospective, comparative, longitudinal feasibility study
Source: BMC Pregnancy Childbirth. 2022 Jul 6;22:546. doi: 10.1186/s12884-022-04862-1 (PMC9258469; doi:10.1186/s12884-022-04862-1)
Supplement: Supplementary file 3 — Additional file 3. [file 12884_2022_4862_MOESM3_ESM.docx]

**Table S3. Selected variables associated with response/nonresponse at six months postpartum (fathers/non-birth-giving mothers)**

|  | **Group A. Preterm group**  N = 21 | | | **Group B. Full-born group****  N = 61 | | |
| --- | --- | --- | --- | --- | --- | --- |
|  | Completers  n = 6 (28.6%) | Dropouts  n = 15 (71.4%) | p-value | Completers  n = 31 (50.8%) | Dropouts  n = 30 (49.0%) | p-value |
|  | **n (%)** | **n (%)** |  | **n (%)** | **n (%)** |  |
| Infant birthweight | 6 (28.6) | 15 (71.4) | N/A* | 31 (50.8) | 30 (49.0) | 1.0 |
| Infant’s gestational age level | 6 (28.6) | 15 (71.4) | N/A* | 31 (50.8) | 30 (49.0) | N/A* |
| Parity | 6 (28.6) | 15 (71.4) | 0.5 | 31 (50.8) | 30 (49.0) | N/A* |
| Fatigue | 6 (28.6) | 15 (71.4) | 0.6 | 31 (50.8) | 30 (49.0) | 0.7 |
| Depression | 6 (28.6) | 15 (71.4) | 0.5 | 31 (50.8) | 30 (49.0) | 0.8 |
| Insomnia | 6 (28.6) | 15 (71.4) | 0.2 | 31 (50.8) | 30 (49.0) | 0.8 |
| Education | 6 (28.6) | 15 (71.4) | N/A* | 31 (50.8) | 30 (49.0) | N/A* |
| Income | 6 (28.6) | 15 (71.4) | N/A* | 31 (50.8) | 30 (49.0) | N/A* |
| Employment status | 6 (28.6) | 15 (71.4) | 0.5 | 31 (50.8) | 30 (49.0) | 1.0 |
| Ethnicity | 6 (28.6) | 15 (71.4) | 0.4 | 31 (50.8) | 30 (49.0) | N/A* |
|  | **Median (range)** | **Median (range)** |  | **Median (range)** | **Median (range)** |  |
| Body mass index | 26.5 (11.9) | 23.7 (13.3) | 0.5 | 26.1 (15.2) | 26.8 (11.7) | 0.3 |
| Age | 32.0 (7.0) | 32.0 (8.0) | 0.7 | 34.0 (18.0) | 32.5 (17.0) | 0.5 |
| HRQoL (physical) | 52.9 (10.9) | 46.7 (14.7) | 0.5 | 53.8 (24.3) | 52.6 (15.0) | 0.7 |
| HRQoL (mental) | 47.0 (11.2) | 50.0 (30.2) | 0.5 | 52.2 (28.8) | 47.9 (35.7) | 0.5 |
| Stress | 0.2 (0.2) | 0.3 (0.5) | 0.6 | 0.3 (0.7) | 0.4 (0.8) | 0.1 |
| Social support | 1.3 (0.8) | 1.2 (0.6) | 0.3 | 1.6 (3.6) | 2.0 (3.1) | 0.7 |
| Self-efficacy | 16.5 (8.0) | 16.0 (9.0) | 0.9 | 17.0 (9.0) | 16.0 (8.0) | 0.4 |

* N/A = N/A not analysed due to too small sample size/too limited statistical power.

** Including non-birth-giving mothers. Range = max-min value
