# Supplementary material for: Sleep patterns and psychosocial health of parents of preterm and full-born infants: a prospective, comparative, longitudinal feasibility study
Source: BMC Pregnancy Childbirth. 2022 Jul 6;22:546. doi: 10.1186/s12884-022-04862-1 (PMC9258469; doi:10.1186/s12884-022-04862-1)
Supplement: Supplementary file 4 — Additional file 4. [file 12884_2022_4862_MOESM4_ESM.docx]

**Table S4. Selected variables associated with response/nonresponse at 12 months postpartum (mothers)**

|  | **Group A Preterm group**  **N = 17** | | | **Group B Full- born group**  **N = 60**** | | |
| --- | --- | --- | --- | --- | --- | --- |
|  | Completers  n = 10 (58.8%) | Dropouts  n = 7 (41.1%) | p-value | Completers  n = 30 (50.0%) | Dropouts  n = 30 (50.0%) | p-value |
|  | **n (%)** | **n (%)** |  | **n (%)** | **n (%)** |  |
| Infant’s birthweight | 10 (58.8) | 7 (41.1) | N/A* | 30 (50.0) | 30 (50.0) | 0.3 |
| Infant’s gestational age level | 10 (58.8) | 7 (41.1) | N/A* | 30 (50.0) | 30 (50.0) | N/A* |
| Parity | 10 (58.8) | 7 (41.1) | 1.0 | 30 (50.0) | 30 (50.0) | N/A* |
| Fatigue | 10 (58.8) | 7 (41.1) | 1.0 | 30 (50.0) | 30 (50.0) | 0.7 |
| Depression | 10 (58.8) | 7 (41.1) | 0.5 | 30 (50.0) | 30 (50.0) | 1.0 |
| Insomnia | 10 (58.8) | 7 (41.1) | 0.3 | 30 (50.0) | 30 (50.0) | 0.6 |
| Education | 10 (58.8) | 7 (41.1) | N/A* | 30 (50.0) | 30 (50.0) | N/A* |
| Income | 10 (58.8) | 7 (41.1) | N/A* | 30 (50.0) | 30 (50.0) | N/A* |
| Employment status | 10 (58.8) | 7 (41.1) | 1.0 | 30 (50.0) | 30 (50.0) | 0.1 |
| Ethnicity | 10 (58.8) | 7 (41.1) | 1.0 | 30 (50.0) | 30 (50.0) | N/A* |
|  | **Median (range)** | **Median (range)** |  | **Median (range)** | **Median (range)** |  |
| Body mass index | 26.9 (19.8) | 24.0 (13.9) | 0.4 | 23.6 (14.2) | 25.0 (18.4) | 0.3 |
| Age | 29.0 (6.0) | 31.0 (9.0) | 0.2 | 32.0 (15.0) | 30.0 (20.0) | **0.0** |
| HRQoL (physical) | 50.6 (18.0) | 48.1 (43.7) | 0.5 | 51.5 (40.4) | 49.2 (23.4) | 0.3 |
| HRQoL (mental) | 50.6 (18.2) | 43.0 (27.2) | 0.2 | 47.7 (40.4) | 51.7 (45.0) | 0.9 |
| Stress | 0.2 (0.2) | 0.3 (0.5) | 0.7 | 0.3 (0.6) | 0.3 (0.7) | 0.9 |
| Social support | 1.3 (0.8) | 1.0 (1.5) | 0.4 | 1.5 (2.5) | 1.6 (3.5) | 0.4 |
| Self-efficacy | 15.0 (4.0) | 14.0 (11.0) | 0.7 | 17.0 (13.0) | 16.0 (13.0) | 0.2 |

* N/A = N/A not analysed due to too small sample size/too limited statistical power.

** Including non-birth-giving mothers. Range = max-min value
