# Supplementary material for: Sleep patterns and psychosocial health of parents of preterm and full-born infants: a prospective, comparative, longitudinal feasibility study
Source: BMC Pregnancy Childbirth. 2022 Jul 6;22:546. doi: 10.1186/s12884-022-04862-1 (PMC9258469; doi:10.1186/s12884-022-04862-1)
Supplement: Supplementary file 5 — Additional file 5. [file 12884_2022_4862_MOESM5_ESM.docx]

**Table S5. Selected variables association with response/ nonresponse at 12 months postpartum (fathers)**

|  | **Group A Preterm group**  **N = 17** | | | **Group B Full-born group**  **N = 60**** | | |
| --- | --- | --- | --- | --- | --- | --- |
|  | Completers  n = 8 (47.0%) | Dropouts  n = 9 (52.9%) | p-value | Completers  n = 31 (53.4%) | Dropouts  n = 29 (46.5%) | p-value |
|  | **n (%)** | **n (%)** |  | **n (%)** | **n (%)** |  |
| Infant’s birthweight | 8 (47.0) | 9 (52.9) | N/A* | 31 (53.4) | 29 (48.0) | 0.3 |
| Infant’s gestational age level | 8 (47.0) | 9 (52.9) | N/A* | 31 (53.4) | 29 (48.0) | N/A* |
| Parity | 8 (47.0) | 9 (52.9) | N/A* | 31 (53.4) | 29 (48.0) | N/A* |
| Fatigue | 8 (47.0) | 9 (52.9) | 0.6 | 31 (53.4) | 29 (48.0) | 0.7 |
| Depression | 8 (47.0) | 9 (52.9) | 1.0 | 31 (53.4) | 29 (48.0) | 0.7 |
| Insomnia | 8 (47.0) | 9 (52.9) | 0.5 | 31 (53.4) | 29 (48.0) | 1.0 |
| Education | 8 (47.0) | 9 (52.9) | N/A* | 31 (53.4) | 29 (48.0) | N/A* |
| Income | 8 (47.0) | 9 (52.9) | N/A* | 31 (53.4) | 29 (48.0) | N/A* |
| Employment status | 8 (47.0) | 9 (52.9) | 0.1 | 31 (53.4) | 29 (48.0) | 0.6 |
| Ethnicity | 8 (47.0) | 9 (52.9) | N/A* | 31 (53.4) | 29 (48.0) | N/A* |
|  | **Median (range)** | **Median (range)** |  | **Median (range)** | **Median (range)** |  |
| Body mass index | 23.8 (9.4) | 30.6 (10.7) | **0.0** | 26.4 (13.3) | 26.7 (13.5) | 1.0 |
| Age | 31.0 (9.0) | 33.5 (4.0) | **0.0** | 34.0 (19.0) | 31.5 (15.0) | **0.0** |
| HRQoL (physical) | 52.4 (13.9) | 47.8 (14.7) | 0.2 | 53.0 (21.0) | 55.3 (17.5) | 0.1 |
| HRQoL (mental) | 47.9 (24.5) | 51.6 (25.0) | 0.3 | 49.5 (35.0) | 53.2 (30.7) | 0.5 |
| Stress | 0.2 (0.3) | 0.3 (0.5) | 0.4 | 0.3 (0.8) | 0.3 (0.7) | 0.8 |
| Social support | 1.3 (0.8) | 1.3 (0.6) | 0.7 | 2.0 (3.5) | 1.6 (2.3) | 0.3 |
| Self-efficacy | 16.5 (8.0) | 16.0 (9.0) | 0.9 | 16.5 (8.0) | 16.5 (9.0) | 1.0 |

* N/A = N/A not analysed due to too small sample size/too limited statistical power.

** Including non-birth-giving mothers. Range = max-min value
